# Effect of cervical mucus aspiration before embryo transfer on pregnancy rate

**Published:** 2013-10

**Authors:** Soghra Rabiee, Roya Kaboodmehri, Mohammad Fallah, Mahnaz Yavangi, Marzieh Sanouei Farimani

**Affiliations:** 1 *Department of Obstetrics and Gynecology, Fatemieh Women Hospital, Hamadan University of Medical Sciences, Hamadan, Iran.*; 2 *Department of Parasitology and Mycology, School of Medicine, Hamadan University of Medical Sciences, Hamadan, Iran.*

Embryo transfer and its related factors received little clinical attention and had been, until recently, the most inefficient step in in-vitro fertilization (IVF). Factors which appear to influence implantation rates are: contamination of the catheter tip with cervical bacteria, stimulation of uterine contractions during the procedure, the type of catheter, ultrasound guidance during the transfer, the position of the embryos in the uterine cavity and perhaps cervical mucus (1-4). Easy and atraumatic transfer is essential for successful implantation and the embryos need to be placed in the middle of the cavity, away from the fundus (5). The goal of trans-cervical embryo transfer is to non-traumatic deliver the embryo to an optimal intra uterine location for implantation. Cervical canal mucus may cover the catheter tip and it can be a source of bacterial contamination of uterine cavity. Therefore, aim of this study was evaluation of effect of removal of cervical mucus on clinical pregnancy rate. This study was carried out as a randomized controlled trial. Randomization was done for stratification of age, method of treatment and cause of infertility. 

A total of 120 women (18-35 years) with male factor infertility, was undergoing IVF cycles with long protocol, divided to two groups: 60 infertile women as cases (group A) that cervical mucus was aspirated and 60 women as controls (group B), without aspiration. In both groups scrub was done by normal saline. Aspiration of cervical mucus was performed by Mucat catheter just before embryo transfer in case group. Embryo transfer was done after 36 hours of puncture. Bed rest for all women after embryo transfer was 1 hours. Primary outcome and pregnancy defined as: positive βhCG 12 days after embryo transfer. 

The mean age of group A was 29.93±5.04 years, and in the group B was 29.03±4.5 years (p>0.05). The mean duration of infertility in group A was 7.6±5.6 years and in group B the mean duration of infertility was 5.5±3.2 years (p>0.05). The frequencies of previous IVF in group A and B was 38.3%, and 28.13% respectively (p>0.05, OR=1.64 in group A, OR=2.64 in group B). There was no significant difference between two groups in terms of number of transferred embryos statistically (p=0.06). The quality of transferred embryo in group A was as following: grade a 67.7%, grade b 16.7%, grade c 6.7%, and in group B was: grade a 85% and grade b 15%, and two groups were also no significantly different (p>0.05). Contact bleeding was happened in 1.7% of group A and 3.3% of group B. The rate of pregnancy (positive βhCG) in the group A was 11.7% (n=7), however, in the group B was 16.7% (n=10) and two groups had not significant difference statistically (p>0.05, OR=0.66). This study indicates that, removal of cervical mucus during embryo transfer (ET) has no positive effect on the pregnancy rate. However, according to some reports, removal of cervical mucus during ET had been postulated to increase the pregnancy and implantation rates by not interfering with embryo implantation (6). Some researchers suggested that, this is a time- consuming procedure that may increase the incidence of difficult transfers by removing the naturally lubricant mucus. 

In addition, any cervical manipulation at the time of embryo transfer may cause unwarranted uterine contraction. Several studies have shown a correlation between cervical mucus aspiration and increase pregnancy rates (4, 7). According to the study of Yazd University of Medical Sciences, cervical mucus aspiration with insulin syringe before embryo transfer can increase the pregnancy rate (8). According to some reports the presence of bacterial contamination of catheter tip during embryo is evidently limited and does not significantly affect the cycle outcomes (2). Several studies have shown that cervical mucus aspiration can decrease infection rate with E. coli, Mycoplasma, Uroplasma, Streptococcus B, D, Staphilococcus and increase implantation rate (9). 

In addition to cleaning cervical mucus, other interventions, such as drug prescription (ritodrine for example) also has no significant effect on pregnancy rate (10). Present study showed no positive correlation between this intervention and result of pregnancy outcome; however, total pregnancy rate in both groups was not high. Because of this procedure may increase the incidence of difficult transfer by removing the naturally lubricant mucus and may cause uterine contraction. In conclusion, the data presented in this study suggest that cervical area and uterus environment manipulation before embryo transfer is not recommended.
